# The feeding practices and structure questionnaire: construction and initial validation in a sample of Australian first-time mothers and their 2-year olds

**DOI:** 10.1186/1479-5868-11-72

**Published:** 2014-06-04

**Authors:** Elena Jansen, Kimberley M Mallan, Jan M Nicholson, Lynne A Daniels

**Affiliations:** 1 Institute of Health and Biomedical Innovation, School of Exercise and Nutrition Sciences, Queensland University of Technology, 60 Musk Avenue, Kelvin Grove, Queensland 4059, Australia; 2 Parenting Research Centre, 232 Victoria Parade, East Melbourne, Victoria 3002, Australia; 3 School of Early Childhood, Faculty of Education, Queensland University of Technology, Brisbane 4059, Australia; 4 Nutrition and Dietetics, Flinders University, Adelaide, South Australia 5001, Australia

**Keywords:** Feeding practices, Structured mealtimes, Control, Responsive feeding, Authoritative feeding, Confirmatory factor analysis, Childhood obesity

## Abstract

**Background:**

Early feeding practices lay the foundation for children’s eating habits and weight gain. Questionnaires are available to assess parental feeding but overlapping and inconsistent items, subscales and terminology limit conceptual clarity and between study comparisons. Our aim was to consolidate a range of existing items into a parsimonious and conceptually robust questionnaire for assessing feeding practices with very young children (<3 years).

**Methods:**

Data were from 462 mothers and children (age 21–27 months) from the NOURISH trial. Items from five questionnaires and two study-specific items were submitted to *a priori* item selection, allocation and verification, before theoretically-derived factors were tested using Confirmatory Factor Analysis. Construct validity of the new factors was examined by correlating these with child eating behaviours and weight.

**Results:**

Following expert review 10 factors were specified. Of these, 9 factors (40 items) showed acceptable model fit and internal reliability (Cronbach’s α: 0.61-0.89). Four factors reflected non-responsive feeding practices: ‘Distrust in Appetite’, ‘Reward for Behaviour’, ‘Reward for Eating’, and ‘Persuasive Feeding’. Five factors reflected structure of the meal environment and limits: ‘Structured Meal Setting’, ‘Structured Meal Timing’, ‘Family Meal Setting’, ‘Overt Restriction’ and ‘Covert Restriction’. Feeding practices generally showed the expected pattern of associations with child eating behaviours but none with weight.

**Conclusion:**

The Feeding Practices and Structure Questionnaire (FPSQ) provides a new reliable and valid measure of parental feeding practices, specifically maternal responsiveness to children’s hunger/satiety signals facilitated by routine and structure in feeding. Further validation in more diverse samples is required.

## Background

What and how parents feed their children shapes early eating habits and consequent risks for excess weight gain and obesity [1-3]. While a range of questionnaires have been developed to assess parental feeding practices, their practical use is limited by lack of conceptual clarity over what is being measured compounded by overlapping and inconsistent item sets and subscales [4,5]. Importantly, very few measures have been validated for use with parents of children under 3 years of age, a life stage when eating habits are established and arguably are the most sensitive to parental feeding practices [6-8]. Together, these limitations hinder attempts to understand how early parental feeding practices influence children’s eating behaviours and thus, restrict opportunities to identify potential avenues for preventing childhood obesity.

Parents’ interactions with their children around food and eating have been conceptualised as a context-specific aspect of broader parenting behaviour [1,9,10]. ‘Parenting’ refers to child-rearing activities which aim to promote and support children’s development [11]. One common approach has been to conceptualise parenting according to relatively enduring ‘styles’ of interaction (i.e., authoritative, authoritarian, permissive and neglectful) [12,13], underpinned by two key behavioural dimensions – the extent to which parents are responsive to their children’s needs and demands (parental ‘responsiveness’), and the extent to which parents set clear limits around their children’s behaviour and consistently ensure compliance (parental ‘demandingness’ or ‘control’) [14]. Children who experience authoritative parenting (high responsiveness, high demandingness) show positive outcomes in health risk behaviours, cognitive ability and socio-emotional competence [15-19]. While there is emerging evidence that an authoritative parenting style may also be protective against childhood obesity [20-25], observed associations are relatively weak. It is likely that parenting behaviours that are specific to their children’s eating have a stronger direct impact on child weight [1,26].

Similar to general parenting, ‘parent feeding practices’ (also referred to as ‘food parenting’ [5,27,28]) have been described in terms of both styles and practices [3,4]. It has been suggested that ‘authoritative feeding’ (the combination of responsive feeding and structure of the meal environment) may promote the development of healthy eating patterns [8,29-34]. Although not explicitly referred to as ‘authoritative feeding’, Satter’s early work in the clinical failure to thrive context [35] and its extension to a broader obesity prevention context (the Trust Model) [33] asserts that healthy eating is promoted by parental responsibility for structuring the feeding environment–the what, when and where of food provision (i.e., ‘demandingness’ characterised in terms of ‘limits’ and ‘structure’ [29,36] rather than ‘control’)–combined with supportive parental responses to children’s cues of hunger and satiety–allowing the child to determine whether and how much to eat (i.e., responsiveness) [35]. Together these behaviours create a predictable, developmentally appropriate feeding environment, which allows children to attend to and recognise internal hunger and satiety cues and to maintain their capacity to self-regulate energy intake [8,29,33].

The concept of authoritative feeding practices provides an inherently plausible and flexible framework for considering how a number of discrete feeding practices may individually or in combination, influence the development of healthy eating in early life. However, no single measure exists that assesses a comprehensive set of relevant dimensions of feeding responsiveness and mealtime structure simultaneously in very young children (<3 years of age) [4,6,27,30,34,37]. The aim of the current study was to construct and evaluate a parsimonious and conceptually robust questionnaire for assessing the parental feeding practices that support development of healthy eating behaviour. Using an existing data set [38,39], we sought to construct the Feeding Practices and Structure Questionnaire (FPSQ) comprising a number of feeding practices scales that would assess conceptually distinct dimensions of responsive feeding (practices that support children’s self-regulation of intake) and appropriate structure and limits (practices that create an environment supportive of healthy eating). The consolidation, construction and validation steps undertaken correspond to the first five of six steps recently proposed by Vaughn et al*. *[27] for the development of a robust measure of parental feeding: (1) clear conceptualisation of what is being measured, (2) systematic development of the item pool, (3) refinement of the item pool, (4) reliability testing, (5) validity testing, and (6) responsiveness or stability testing. The sixth step will be evaluated using forthcoming longitudinal data [38].

## Methods

### Participants and procedure

Data were sourced from participants enrolled in the NOURISH randomised controlled trial (RCT; Australian and New Zealand Clinical Trials Registry Number 12608000056392) conducted from February 2008 to May 2011. NOURISH evaluated an early feeding intervention designed to promote maternal feeding practices that supported healthy child growth [39]. Participants were a consecutive sample of first-time mothers (≥18 years old) recruited through maternity hospitals in Adelaide and Brisbane, who had delivered a healthy term baby (>35 weeks, >2500 g), and had sufficient facility with English to participate in intervention sessions and complete questionnaires. The trial protocol, recruitment and participant characteristics have been described elsewhere [39,40]. Of the 698 mothers randomly allocated to intervention or control group, 467 (67%) completed the self-administered questionnaire at the third assessment time point (child age: 21–27 months), forming the present study sample. Demographic characteristics included child gender, age (months), maternal age (years), BMI (kg/m^2^, measured weight and height), education level and marital status. NOURISH was approved by the Queensland University of Technology Human Research Ethics Committee.

### Item sources

Mothers’ feeding practices were assessed at NOURISH follow-up (child age 2 years) via a self-administered questionnaire in which 89 items from five existing measures were included. These were from (i) the Child Feeding Questionnaire (CFQ) [41], restriction (8 items), pressure to eat (4 items) and monitoring (3 items); (ii) the Caregiver’s Feeding Style Questionnaire (CFSQ) [42], child-centred strategies (7 items) and parent-centred strategies (12 items); (iii) Ogden et al. [43], overt control (4 items) and covert control (5 items); (iv) the Parental Feeding Style Questionnaire (PFSQ) [2], emotional feeding (5 items), instrumental feeding (4 items), control over eating (10 items) and promoting or encouragement to eat (8 items), and (v) Chan et al. [44], managing the feeding environment (5 items) and maternal responses to the child’s refusal of familiar foods (8 items). Two novel items were included to assess Satter’s ‘division of responsibility’ principle [45,46] (see Table 1). For the current study, these 91 items formed the item pool and were considered for *a priori* selection and allocation to the newly conceptualised feeding practices scales.

**Table 1:** **Feeding items by Chan, Magarey and Daniels **[44] **and Satter **[45,46]

**Item**	**Response options**
*By Chan, Magarey and Daniels*	
My child eats main meals with the rest of the family.	(1) A lot of the time
My child eats the same meals as the rest of the family.	(2) Very often
My child sits down when having meals.	(3) Often
My child watches television when having meals.	(4) Sometimes
I cook separate meals for my child.	(5) Hardly ever
When your child refuses food they usually eat, do you…	(1) Never
…insist your child eats it?	(2) Not often
…offer another food that (s)he usually likes?	(3) Sometimes
…encourage to eat by turning mealtime into a game (e.g., pretending loaded spoon is an aeroplane)?	(4) Often
	(5) Most of the time
…encourage to eat by offering a food reward (e.g., dessert)?	
…encourage to eat by offering a reward other than food?	
…offer no food until next usual meal or snack time?	
…accept that your child may not be hungry and take the food away?	
…punish your child in some way?*	
*By Satter*	
Who decides what food your child eats–you or your child?	(1) You only
Who decides how much food your child eats–you or your child?	(2) Mostly you
	(3) You & your child equally
	(4) Mostly your child
	(5) Your child only

*This item was added to the NOURISH questionnaire and not originally developed in Chan et al. [44].

### Item consolidation, factor identification, specification and validation

**Figure 1:**
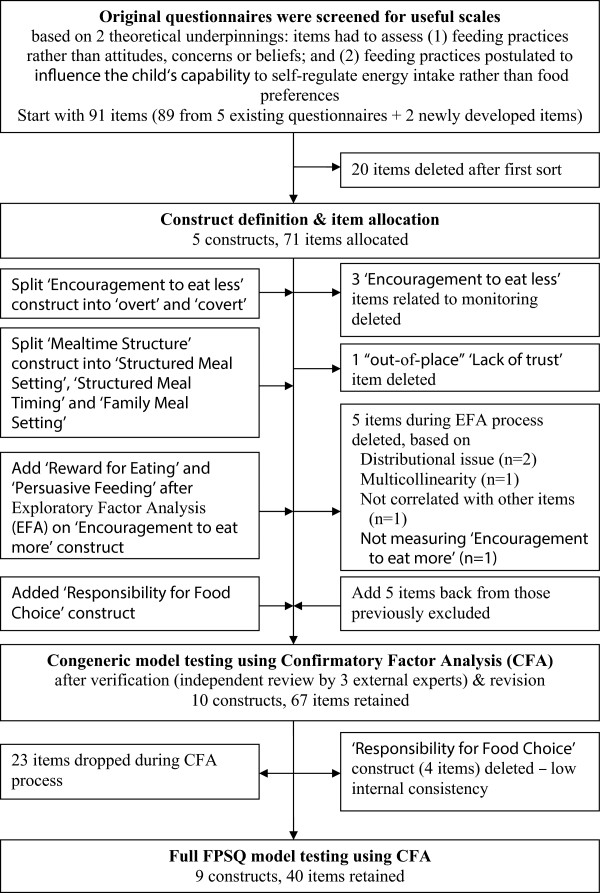
Overview of the number of factors and items at each step of the measurement development process.

Figure 1 provides a flow chart of the steps taken to construct and validate the questionnaire.

### Construct definition and item allocation

The first step in measurement construction involved *a priori* definition of the constructs of authoritative feeding practices, followed by initial assignment of items to each construct (see Figure 1). We proposed five constructs as capturing the key components of ‘authoritative feeding’. Four of these constructs reflected non-responsive feeding practices that could interfere with or override the child’s self-regulatory capabilities: (1) practices that indicated a lack of trust in child’s capabilities to self-regulate intake; (2) using food unrelated to appetite; (3) encouragement to eat more; and (4) encouragement to eat less. The fifth construct was structured mealtime environment and choice (‘Mealtime structure’). Twenty items were judged as not aligning with any of these five constructs and were excluded from further consideration. The remaining 71 items were each assigned to one of the constructs.In the second step, the five proposed constructs and their items were independently reviewed by three external experts. Based on expert feedback, two constructs were divided into five more tightly defined constructs. ‘Encouragement to eat less’ was redefined into covert and overt restriction. ‘Mealtime structure’ was redefined into structure related to setting, to timing and to family meals. For the ‘encouragement to eat more’ construct which initially comprised 25 items, Exploratory Factor Analysis (EFA; in SPSS, factor extraction predominately based on scree plot) was performed to identify statistically viable sub-factors and remove poorly loading items (see Figure 1). One additional construct was added that sought to capture practices that reflected parental responsibility for food choice (‘Responsibility for Food Choice’), with four relevant items added in from the list of previously excluded items.

To ensure clear and unambiguous factor labels, relevant items were reverse coded so that higher scores reflected a higher endorsement of the practice indicated in the label (see Table 2). The final measure taken forward for construct validation comprised 67 items assessing 10 feeding constructs: Distrust in Appetite (8 items), Reward for Behaviour (10 items), Reward for Eating (7 items), Persuasive Feeding (13 items), Covert Restriction (5 items), Overt Restriction (6 items), Structured Meal Setting (5 items), Structured Meal Timing (5 items), Family Meal Setting (3 items), and Responsibility for Food Choice (5 items).

**Table 2:** The Feeding Practices and Structure Questionnaire (FPSQ)–9 factors and 40 items

**Factor**	**Item name**		**Content**
Distrust in appetite	1	DA1	If I did not guide or regulate my child’s eating, (s)he **would eat much less** than (s)he should.^a, 1^
	2	DA2	How often are you **firm about how much** your child should eat?^b, 2^
	3	DA3*	**Who decides** how much food your child eats – you or your child?^c^
	4	DA4*	When your child refuses food they usually eat, do you **accept that** your **child** may **not** be **hungry** and take the food away?^b, 3^
Reward for behaviour	5	RB1	I offer **sweet foods** (lollies, ice-cream, cake, pastries) to my child **as a reward** for good behaviour.^a, 1^
	6	RB2	I offer my child his/her **favourite foods** in exchange **for good behaviour**.^a, 1^
	7	RB3	In order to get my child **to behave** him/herself I **promise** him/her **something to eat**.^b, 4^
	8	RB4	I **reward** my child **with something to eat** when (s)he is well behaved.^b, 4^
	9	RB5	I give my child something to **eat to** make him/her **feel better when** (s)he is feeling **upset**.^b, 4^
	10	RB6	I give my child something to **eat to** make him/her **feel better when** (s)he has been **hurt**.^b, 4^
Reward for eating	11	RE1	…do you **promise** the child something other than food **if** (s)he **eats** (for example, “If you eat your beans, we can go to the park”)?^b, 5^
	12	RE2	When your child refuses food they usually eat, do you encourage to eat by **offering a reward other than food**?^b, 3^
	13	RE3	…do you encourage the child to eat something by **using food as a reward** (for example, “If you finish your vegetables, you will get some fruit)?^b, 5^
	14	RE4	When your child refuses food they usually eat, do you encourage to eat by **offering a food reward** (e.g., dessert)?^b, 3^
	15	RE5	I use **desserts as a bribe** to get my child to eat his/her main course.^b, 4^
	16	RE6	…do you warn the child that you will **take a food away** if the child doesn’t eat (for example, “If you don’t finish your vegetables, you won’t get fruit”)?^b, 5^
Persuasive feeding	17	PF1	If my child says “I’m not hungry” I **try to** get him/her to **eat anyway**.^a, 1^
	18	PF2	When your child refuses food they usually eat, do you **insist your child eats** it?^b, 3^
	19	PF3	I **praise** my child if (s)he eats what I give him/her.^b, 4^
	20	PF4	…do you **reason** with the child to get him/her to eat (for example, “Milk is good for your health because it will make you strong”)?^b, 5^
	21	PF5	…do you **tell** the child to eat something on the plate (for example, “Eat your beans”)?^b, 5^
	22	PF6	…do you say something to **show** your **disapproval** of the child for not eating?^b, 5^
Covert restriction	23	CR1	How often do you **avoid** going with your child to cafes or restaurants which **sell unhealthy foods**?^b, 2^
	24	CR2	How often do you **avoid buying lollies and snacks** e.g., potato chips and bringing them into the house?^b, 2^
	25	CR3	How often do you **not buy foods that you would like** because you do not want your children to have them?^b, 2^
	26	CR4	How often do you **avoid buying biscuits and cakes** and bringing them into the house?^b, 2^
Overt restriction	27	OR1	I have to be sure that my child does **not eat too many sweet foods** (lollies, ice-cream, cake or pastries).^a, 1^
	28	OR2	I have to be sure that my child does **not eat too much** of his/her **favourite foods**.^a, 1^
	29	OR3	I intentionally **keep some foods out of** my child’s **reach**.^a, 1^
	30	OR4	If I did not guide or regulate my child’s eating, (s)he **would eat too many junk foods**.^a, 1^
Structured meal setting	31	SMS1*	I allow my child to **wander** around **during a meal**.^b, 4^
	32	SMS2	I insist my child **eats** meals **at the table**.^b, 4^
	33	SMS3	How often are you **firm about where** your child should eat?^b, 2^
	34	SMS4	My child **sits down** when having meals.^b, 3^
Structured meal timing	35	SMT1*	I let my **child decide when** (s)he would like to have her meal.^b, 4^
	36	SMT2	**I decide when** it is time for my child to have a **snack**.^b, 4^
	37	SMT3	**I decide** the times **when** my child eats his/her **meals**.^b, 4^
Family meal setting	38	FMS1	My child eats **main meals with** the rest of the **family**.^b, 3^
	39	FMS2	My child eats the **same meals as** the rest of the **family**.^b, 3^
	40	FMS3*	I **cook separate meals** for my child.^b, 3^

*Item is reverse coded.

^a^Response options: (1) Disagree, (2) Slightly disagree, (3) Neutral, (4) Slightly agree, (5) Agree.

^b^Response options: (1) Never, (2) Rarely, (3) Sometimes, (4) Often, (5) Always.

^c^Response options: (1) You only, (2) Mostly you, (3) You and your child equally, (4) Mostly your child, (5) Your child only.

^1^From Child Feeding Questionnaire by Birch et al. [41].

^2^From Ogden et al.’s [43] measure of overt and covert control.

^3^From Chan et al.’s [44] measure of feeding environment management and responses to food refusal.

^4^From Parental Feeding Style Questionnaire by Wardle et al. [2].

**Figure 2:**
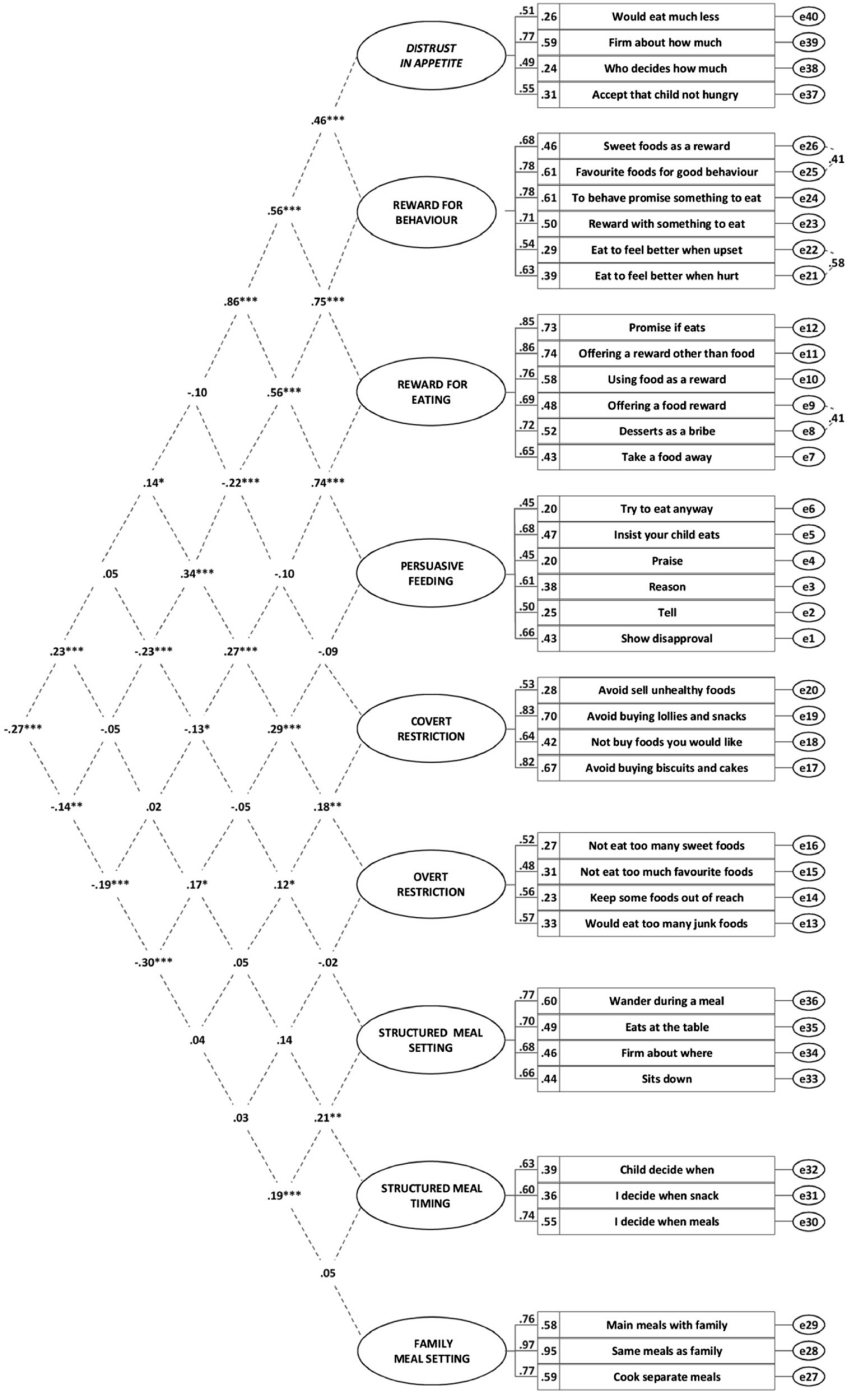
Full FPSQ model with 9 factors and 40 items, showing factor-factor correlations, standardised factor loadings, squared multiple correlations and correlations of error terms.

^5^From Caregiver’s Feeding Styles Questionnaire by Hughes et al. [42].Note: Key words presented in bold are listed on the model in Figure 2, rather than the full items.

The original response options for items 1, 2, 3 and 7 were: (1) A lot of the time, (2) Very often, (3) Often, (4) Sometimes, (5) Hardly ever; for items 22, 30, 32 and 36: (1) Never, (2) Not often, (3) Sometimes, (4) Often, (5) Most of the time; and for items 29, 31, 34, 38, 39 and 40: (1) Never, (2) Rarely, (3) Sometimes, (4) Most of the time, (5) Always.

### Statistical construct specification – congeneric models

Confirmatory Factor Analysis (CFA) using maximum likelihood estimation was performed in AMOS 19.0. Statistical validation of the newly formed, theoretically-based feeding practices factors included examination and, where necessary, re-specification of the individual congeneric models (i.e. one-dimensional models; all items are expected to load on one latent variable) to create the best performing, most parsimonious item sets for each factor. Initial model specifications included fixing one regression weight per factor to 1. Performance of the ‘Family Meal Setting’ congeneric model could not be tested alone as it only consisted of three items. As a solution, this model was tested simultaneously with two other congeneric models (i.e. ‘Structured Meal Setting’ and ‘Structured Meal Timing’) that were initially hypothesised to measure the same construct (i.e. ‘Mealtime structure’).

A range of goodness-of-fit indices were used to evaluate model fit and compare alternative models [47]. Fit indices and their acceptable cut-offs included the normed chi-square (χ^2^/df; values between 1.0–2.0), Comparative Fit Index (CFI; >0.90), Tucker-Lewis Index (TLI; >0.90), Root Mean-Square Error of Approximation (RMSEA; <0.08), and the Akaike Information Criterion (AIC; the smaller the more parsimonious) [48,49]. Model fit was seen as achieved if the majority of fit-indices met the ‘acceptable’ cut-off criteria.

As the goal of fitting the congeneric models was to identify the strongest and most parsimonious set of items for each feeding construct, model re-specification was undertaken if model fit was not achieved (i.e. *post hoc* modification to improve model fit). Item performance was evaluated by considering: item-factor loadings, squared multiple correlations (SMC), response distributions, standardised residuals and modification indices. Items identified as having poor measurement properties were removed. Decisions to add an error covariance were informed by scrutiny of empirical indicators (i.e. modification indices and standardised residual matrix provided by AMOS) and other considerations such as theoretical relatedness and/or similar item wording and response format.

Internal consistency of all newly formed factors was determined using Cronbach’s alpha and coefficient *H*. Factors with a Cronbach’s α < 0.6 were excluded from further consideration on the basis of poor internal reliability [50].

### Statistical construct specification – full model

In the final step, the full measurement model (combination of all valid congeneric models; see Figure 2) was evaluated. As this analysis involved confirming the factorial validity of the FPSQ, *post hoc* modifications were not considered. The same goodness-of-fit indices were used as for the assessment of the congeneric models.

### Predictive validity^a^

Pearson’s correlations between the factors of the FPSQ (weighted composite scores) and children’s eating behaviour and weight (also collected at child age 2 years) were calculated as measure of predictive validity. It was predicted that adaptive eating behaviours and lower weight would be associated with lower and higher scores on the non-responsive and structure/limits factors, respectively. Child eating behaviours were assessed using the 35-item Children’s Eating Behaviour Questionnaire (CEBQ) [51], consisting of eight subscales: satiety responsiveness, slowness in eating, food fussiness, food responsiveness, emotional under-eating, emotional over-eating, enjoyment of food, and desire to drink. The eating behaviour scales ‘Satiety Responsiveness’ and ‘Slowness in Eating’ were combined as suggested by previous research [51,52]. Thus mean scores on 7 scales were calculated with a possible range of 1 (lowest) to 5 (highest). The CEBQ has previously shown good psychometric properties (e.g. concurrent validity, internal consistency and test-retest reliability) [51,53] and has been validated in the control group of the present sample (i.e. the factor structured was confirmed and all susbscales showed good internal reliability with Cronbach’s alpha values between .73 to .91) [52]. Child weight and height were measured by trained study staff [39] and converted to child weight-for-age z-scores (WAZ) using the WHO Anthro version 3.0.1 and macros [54].

### Data

The sample for this study was restricted to the 462 mothers who had less than 20% missing data on any of the newly proposed maternal feeding practices scales. To facilitate analysis in AMOS, remaining missing data on the feeding practices items were imputed using the Expectation Maximization (EM) method. For correlation analysis, cases with missing data were excluded pairwise. Additional data preparation included assessment of multivariate normality (Mardia’s normalised estimate of multivariate kurtosis >5.0) [55] and multivariate outliers. No influential data points were identified with all Cook’s distance values <1. Due to non-normality, the bootstrapping approach and Bollen-Stine bootstrapped chi-square were applied in all analyses in AMOS.

## Results

### Sample characteristics

Mothers (n = 462) had a mean age of 33 (SD = 5; range: 20–48) years at the time of data collection, were well educated (65% with university degree), the majority lived with a partner (97%), and around half (51%) were either overweight or obese (BMI >25; excluding those currently pregnant, n = 131). Approximately half of the children were girls (52%) with mean age 24 (SD = 1; range: 21–27) months and mean WAZ 0.7 (SD = 1.0; range: -3.0–3.5).

### Congeneric models

Following modifications (between 1 and 7 per model), 9 of the 10 congeneric models tested showed acceptable fit: Distrust in Appetite (4 items), Reward for Behaviour (6 items), Reward for Eating (6 items), Persuasive Feeding (6 items), Covert Restriction (4 items), Overt Restriction (4 items), Structured Meal Setting (4 items), Structured Meal Timing (3 items), Family Meal Setting (3 items) (see Table 2). The ‘Responsibility for Food Choice’ (4 items) factor was excluded from further consideration due to poor internal consistency (Cronbach’s α = 0.57). During the model fitting process for the proposed scales 23 items were removed due to poor measurement properties. For the ‘Reward for Behaviour’ model two error covariances (i.e., between items RB1 + RB2 and RB5 + RB6) were added and for the ‘Reward for Eating’ model one error covariance was added (i.e., between items RE4 + RE5).

### Full model – 9-factor Feeding Practices and Structure Questionnaire (FPSQ)

Figure 2 shows the 9-factor model of the FPSQ. Model specifications included correlations between the nine factors and three error covariances (mentioned above). Factors with their respective items and response options are presented in Table 2. Descriptive statistics and measures of internal consistency of the 9 subscales are presented in Table 3. The 9-factor model showed acceptable fit: χ^2^/df = 1.81 was within the desirable range and values of RMSEA = .04, CFI = .92 and TLI = .91 reached acceptable levels. All items had significant standardised factor loadings above 0.4 (i.e., item validity) and a reasonable proportion of variance within each individual item was explained by the respective factor on which it loaded (SMC ≥0.20; i.e., item reliability).

**Table 3:** Descriptive statistics, measures of internal consistency and goodness-of-fit indices for the 9 newly formed feeding practices scales – n = 462 Australian first-time mothers of 24-month-olds

**Factor**	**No. of items**	**Unweighted composite scores**		**Weighted composite scores**		**Reliability**		**Goodness-of-fit indices**			
		**Observed range**	**Mean (SD)**	**Observed range**	**Mean (SD)**	**Coefficient *H***	**Cronbach’s α**	**χ**^**2**^**/df**	**RMSEA**	**CFI**	**TLI**
Distrust in appetite	4	1.00-4.25	2.33 (0.73)	1.00-4.44	2.42 (0.75)	0.72	0.63	4.26	.08	.98	.93
Reward for behaviour	6	1.00-4.33	1.70 (0.69)	1.00-4.43	1.66 (0.68)	0.89	0.86	3.26	.07	.99	.97
Reward for eating	6	1.00-4.83	1.67 (0.70)	1.01-4.85	1.70 (0.74)	0.91	0.89	3.29*	.07	.99	.98
Persuasive feeding	6	1.00-4.50	2.52 (0.67)	1.00-4.29	2.38 (0.68)	0.76	0.73	2.02*	.05	.98	.97
Covert restriction	4	1.00-5.00	3.19 (0.86)	1.00-5.00	3.26 (0.91)	0.84	0.80	2.79*	.06	.99	.98
Overt restriction	4	1.00-5.00	3.38 (0.90)	1.00-5.00	3.43 (0.90)	0.62	0.61	1.57*	.04	.99	.98
Structured meal setting	4	1.75-5.00	4.08 (0.67)	1.63-5.00	4.05 (0.68)	0.80	0.79	2.48	.06	.97	.96
Structured meal timing	3	2.00-5.00	3.86 (0.60)	1.94-5.00	3.90 (0.60)	0.70	0.68				
Family meal setting	3	1.00-5.00	3.93 (1.09)	1.00-4.95	3.88 (1.17)	0.96	0.87				

Note: The possible range is 1 to 5 for each factor.

Goodness-of-fit for the ‘Structured Meal Setting’, ‘Structured Meal Timing’ and ‘Family Meal Setting’ factors was assessed simultaneously because of the low number of items for 2/3 of these congeneric models.

*The congeneric model was non-significant (i.e., p > 0.05), based on the Bollen-Stine bootstrapped chi-square.

Factor-factor correlations were examined to explore whether the associations between subscales corresponded to the two overarching concepts of non-responsive feeding and structuring of the meal environment (see Figure 2). The six strongest factor-factor correlations (all *r* > 0.45) were between ‘Distrust in Appetite’, ‘Reward for Behaviour’, ‘Reward for Eating’ and ‘Persuasive Feeding’, and these factors showed a consistent pattern of correlations with two other factors: all were significantly positively associated with ‘Overt Restriction’ (*r* = 0.14 to 0.34) and significantly negatively associated with ‘Family Meal Setting’ (*r* = -0.14 to -0.30). However, correlations between ‘Covert Restriction’, ‘Overt Restriction’, ‘Structured Meal Setting’, Structured Meal Timing’, and ‘Family Meal Setting’ were predominantly small (*r <* 0.3) [56] and mostly non-significant (6/10).

### Predictive validity

As shown in Table 4, non-responsive and structure-related feeding practices generally showed the expected pattern of associations with child eating behaviours. The four non-responsive feeding practices and Overt Restriction were positively correlated with ‘Fussiness’, ‘Food Responsiveness’, ‘Emotional Eating (over- and under-eating)’ and ‘Desire to Drink’. Persuasive Feeding and Reward for Eating were also negatively correlated with ‘Enjoyment of Food’. As predicted, Structured Meal Setting and Family Meal Setting were positively correlated with ‘Enjoyment of Food’, and negatively correlated with ‘Emotional Eating (over- and under-eating)’ and ‘Fussiness’. Covert Restriction was not significantly correlated with child eating, while Structured Meal Timing was weakly, positively correlated with emotional undereating (*r* = 0.093, p = 0.046). Four unexpected correlations were found with the combined factor ‘Satiety Responsiveness & Slowness in Eating’: Reward for Eating, Persuasive Feeding and Overt Restriction were positively correlated while Structured Meal Setting was negatively correlated. No significant correlations were found between maternal feeding practices and child weight-for-age z-score (Table 4).

**Table 4:** Correlations between feeding practices (weighted composite scores), eating behaviours (mean scores) and child WAZ measured at child age 2 years

	**Satiety responsiveness & slowness eating**	**Fussiness**	**Food responsiveness**	**Enjoyment food**	**Emotional undereating**	**Emotional overeating**	**Desire drink**	**Child weight-for-age z-score**
	**(n = 461)**	**(n = 461)**	**(n = 461)**	**(n = 461)**	**(n = 461)**	**(n = 460)**	**(n = 461)**	**(n = 458)**
Distrust in appetite	-.081	.139**	.153**	-.085	.097*	.225***	.162***	.017
Reward for behaviour	.030	.162***	.339***	-.088	.222***	.386***	.116*	.028
Reward for eating	.101*	.286***	.193***	-.202***	.205***	.287***	.119*	.021
Persuasive feeding	.108*	.266***	.181***	-.173***	.260***	.263***	.197***	-.034
Covert restriction	.025	.005	-.016	.016	.047	-.047	-.020	-.035
Overt restriction	.173***	.132**	.258***	-.074	.180***	.167***	.119*	-.033
Structured meal setting	-.162***	-.142**	-.032	.245***	-.153**	-.136**	-.063	.059
Structured meal timing	-.045	-.048	.012	.074	.093*	-.044	-.050	-.021
Family meal setting	-.014	-.397***	-.017	.286***	-.154**	-.126**	-.087	-.026

*For p < .05, **for p < .01 and ***for p < .001.

## Discussion

This paper describes the construction and validation of the FPSQ. The focus is on maternal responsiveness to children’s signals of hunger and satiety facilitated by routine and structure in feeding as key components of authoritative feeding. Three consecutive phases were undertaken to construct the questionnaire and ensure factors were robust in terms of both content and performance. Phase 1 included *a priori* theory-driven selection of pre-existing items and allocation to potential constructs. Decisions were externally verified and revised, resulting in a total of 10 constructs. Phase 2 used an existing data set [38,39] to undertake a sequential procedure in which Confirmatory Factor Analysis was used to evaluate and modify the 10 congeneric models before confirming the final 9-factor model. Through this process the FPSQ showed acceptable overall goodness-of-fit and appropriate item-level validity and reliability. Phase 3 tested the predictive validity of the FPSQ using concurrent measures of child eating behaviours and weight. In general, the pattern of associations with eating behaviours supported the validity of the questionnaire, however no associations with child weight were found.

According to the Trust Model [33,45,57], a structured and consistent eating environment in which the parent provides children with healthy meals/snacks, coupled with parental responsiveness to the children’s cues of hunger and satiety, supports the development of autonomy and self-regulation of energy intake. The FPSQ captures these two components, measured via nine distinct feeding practices based on 40 items and supported by comprehensive CFA. Four of the subscales assessed an inter-correlated set of non-responsive feeding practices (‘Distrust in Appetite’, ‘Reward for Behaviour’, ‘Reward for Eating’ and ‘Persuasive Feeding’). Although ‘Distrust in Appetite’ and ‘Persuasive Feeding’ were very highly correlated (*r* = 0.86), it was decided to provisionally retain both factors in the questionnaire as it is plausible they are conceptually distinct. The items in ‘Persuasive Feeding’ describe direct and explicit responses to specific cues whereas items in ‘Distrust in Appetite’ describe a more general and overarching response. Future research is needed to determine whether one factor should be excluded, or the items combined to form a single factor. The remaining five factors assessed the two types of restriction (‘Covert Restriction’ and ‘Overt Restriction’) and three aspects of structure of the meal environment (‘Structured Meal Setting’, ‘Structured Meal Timing’ and ‘Family Meal Setting’). Although correlations between these five scales were modest, these factors are argued to be conceptually related to the structure and limits component of the Trust Model [33,45,57].

Satter [35], Eneli et al. [33] and more recently Black and Aboud [8] have argued that a predictable schedule of healthy meals/snacks in an environment which limits distractions (e.g., child seated, no television) supports the child to attend to and effectively communicate hunger and satiety cues that enable the parent to provide the prompt, contingent and predictable response that is the hall mark of responsive feeding [30]. However, the predominance of the use of the Child Feeding Questionnaire [41] has meant that in research terms little attention has been paid to the role of a structured meal time environment. The three mealtime structure scales of the FPSQ will enable examination of the contribution of three distinct aspects of mealtime structure (timing, setting and family engagement) to responsive feeding and child eating behaviour and weight outcomes. The results of such research will provide an evidence base for and enable refinement of the commonly promulgated recommendations for structured meal times [58,59].

Child eating behaviours and weight were used here to test the predictive validity of the FPSQ. Overall the pattern of associations between the feeding practices and child eating behaviours were as expected. With the exception of Covert Restriction and Structured Meal Timing, all feeding practices correlated with at least four child eating behaviours. As expected, high levels of non-responsive feeding practices and low levels of structure-related feeding practices were associated cross-sectionally with the potentially maladaptive eating behaviours. The combined factor of ‘Satiety Responsiveness & Slowness in Eating’ showed correlations with four feeding practices with directions that at face value might be considered somewhat surprising. One possible explanation may be that these associations reflect the bidirectional nature of the feeding relationship [30] and are instances of maternal perception and interpretation of child eating behaviour driving feeding practices [60]. A child who ‘fills up easily’, eats slowly or leaves food on the plate might be perceived as a ‘problem eater’ even though these eating behaviours may be positive if they reflect good satiety responsiveness [51]. In response to this potential misinterpretation of satiety responsiveness as poor eating, mothers may be more likely to use coercive practices such as rewards and persuasion, overtly restrict so children do not ‘fill up’ on junk food, and with a focus on getting the child ‘to at least eat something’ may be less inclined to focus on where the child is eating (less structured meal setting). Future validation studies will need to verify these findings and provide evidence of the predictive validity of Covert Restriction and Structured Meal Timing in particular.

No correlations were evident with child weight-for-age z-score. This finding is not surprising, given that most studies in very young children have failed to find a significant association between feeding practices and BMI [61,62]. This is in contrast to studies in older children where at least some, but not all, commonly considered feeding practices are consistently associated with BMI. There are several plausible potential explanations including (i) the predominance of intrauterine versus postnatal factors in early weight gain [63], (ii) the capacity of feeding practices to support resilience to the obesogenic environment may not manifest until the child is older and more autonomous [38] and (iii) the effect sizes of associations between feeding practices, child eating behaviour and chronic energy balance are likely to be small and need to be sustained over a long period to translate into statistically significant differences in weight status. Thus, although concurrent correlations with the more distal outcome ‘child weight’ appear not to support predictive validity of the FPSQ at this young age, the more proximal child outcome ‘eating behaviour’ provided good evidence for validity of the measurement tool.

## Strengths and limitations

Development of the FPSQ advances the field beyond its current predominant, but somewhat ambiguous and narrow focus on control and emphasises a broader, theory-driven conceptualisation of parental feeding in early childhood. Methodological strengths of the research included *a priori* theory-driven decision making throughout the questionnaire construction phases (e.g., item selection, number of modifications made to models), use of a robust and theory-driven validation procedure using the gold standard Confirmatory Factor Analysis, and examination of predictive validity. Our process closely followed that recently recommended by Vaughn et al. [27].

The study also has a number of limitations. Data from both the NOURISH RCT intervention and control groups were combined to ensure a sufficiently large sample size. Justifications for this decision included: (i) intervention and control groups were comparable across a wide range of covariates at baseline (i.e. successful randomisation) [40]; (ii) the purpose of the present analysis was to evaluate internal consistency and factorial validity, rather than differences between the intervention and control group, and (iii) using the whole sample could potentially increase the variance within variables of interest. Another issue was that due to excess items (n = 25) allocated to one proposed construct it was decided to perform Exploratory Factor Analysis on this factor only for data reduction purposes. Ideally an independent sample would have been used for this analysis.

While we have referred to the FPSQ as ‘parental’ feeding measure, it has been developed and validated with mothers of very young children (21–27 months). In addition, all mothers in this study were primigravid and the majority were Caucasian-Australian, well-educated and living in a defacto relationship or married. Further research is needed to examine how well the FPSQ performs in more diverse samples including samples of mothers and fathers with older children and of different ethnic and cultural backgrounds.

## Recommendations for future research

The development and validation work undertaken here establishes the measurement properties of the newly constructed FPSQ. It is now important to test prospective associations between this measure and children’s eating behaviours and weight. As indicated by Vaughn et al. [27], the stability of the measure over time, and its sensitivity to the effects of parent feeding interventions need to be examined. Additionally, construct validity of the FPSQ can be established through verification with observed feeding interactions or established measures of general parenting styles or dimensions (e.g., responsiveness, demandingness).

## Conclusion

Consistent with numerous recent calls [5,6,27,34,37] for more research regarding the validity and reliability between and within existing feeding measures, this study constructed and statistically validated a new early feeding measure–the FPSQ. The FPSQ provides a conceptually-coherent, theoretically-driven and relatively parsimonious measure of feeding practices related to non-responsiveness and mealtime structure, based on pre-existing items, and validated for use in mothers of toddlers. It consolidates the large number of items/scales available and reduces overlap and ambiguity of terminology and constructs (particularly ‘control’). Importantly, it enhances capacity to examine three distinct, eminently modifiable aspects of meal time structure or limits. The validation procedure needs to be replicated and extended in new and diverse samples. Nevertheless, the FPSQ provides those working in the field of early child nutrition and obesity prevention with a comprehensive tool that can be used in assessment of authoritative feeding characterised by maternal responsiveness to children’s signals of hunger and satiety facilitated by routine and structure in feeding.

## Endnote

^a^Although the commonly used term ‘predictive validity’ is used, all analyses include cross-sectional data.

## Abbreviations

FPSQ: Feeding practices and structure questionnaire; RCT: Randomised controlled trial; CFQ: Child feeding questionnaire; CFSQ: Caregiver’s feeding style questionnaire; PFSQ: Parental feeding style questionnaire; BMI: Body mass index; WHO: World health organization; EFA: Exploratory factor analysis; CFA: Confirmatory factor analysis; CFI: Comparative fit index; TLI: Tucker-Lewis index; RMSEA: Root mean-square error of approximation; AIC: Akaike information criterion; SMC: Squared multiple correlations; EM: Expectation maximization.

## Competing interests

The authors declare that they have no competing interests.

## Authors’ contributions

LD and JN were chief investigators for NOURISH and supervised EJ's doctoral project, which centred on the work presented here. EJ wrote the first draft of the manuscript and conducted all analyses for this secondary data analysis. KM mentored the analyses. All authors contributed conceptually to the measure, reviewed, critiqued and approved this manuscript.
